# SMART transfer method to directly compare the mechanical response of water-supported and free-standing ultrathin polymeric films

**DOI:** 10.1038/s41467-021-22473-w

**Published:** 2021-04-20

**Authors:** Luke A. Galuska, Eric S. Muckley, Zhiqiang Cao, Dakota F. Ehlenberg, Zhiyuan Qian, Song Zhang, Simon Rondeau-Gagné, Minh D. Phan, John F. Ankner, Ilia N. Ivanov, Xiaodan Gu

**Affiliations:** 1 Center for Optoelectronic Materials and Devices, School of Polymer Science and Engineering, University of Southern Mississippi, Hattiesburg, MS 39406 USA; 2 Center for Nanophase Materials Sciences, Oak Ridge National Laboratory, Oak Ridge, TN 37830 USA; 3 Department of Chemistry and Biochemistry, University of Windsor, Windsor, ON Canada N9B3P4; 4 Neutron Scattering Division, Oak Ridge National Laboratory, Oak Ridge, TN 37830 USA

**Keywords:** Structural properties, Polymers, Characterization and analytical techniques

## Abstract

Intrinsic mechanical properties of sub-100 nm thin films are markedly difficult to obtain, yet an ever-growing necessity for emerging fields such as soft organic electronics. To complicate matters, the interfacial contribution plays a major role in such thin films and is often unexplored despite supporting substrates being a main component in current metrologies. Here we present the shear motion assisted robust transfer technique for fabricating free-standing sub-100 nm films and measuring their inherent structural–mechanical properties. We compare these results to water-supported measurements, exploring two phenomena: 1) The influence of confinement on mechanics and 2) the role of water on the mechanical properties of hydrophobic films. Upon confinement, polystyrene films exhibit increased strain at failure, and reduced yield stress, while modulus is reduced only for the thinnest 19 nm film. Water measurements demonstrate subtle differences in mechanics which we elucidate using quartz crystal microbalance and neutron reflectometry.

## Abstract (web summary)

Intrinsic mechanical properties of sub-100 nm thin films are markedly difficult to obtain, yet an ever-growing necessity for emerging fields such as soft organic electronics. Here, the authors present a shear motion assisted transfer technique for fabricating free-standing sub-100 nm thin films and measuring their inherent structural–mechanical properties.

## Introduction

The future of coatings, membranes, and organic electronics (thin-film transistors, photovoltaics, sensors, and bioelectronics) relies on a thorough understanding of polymer thin-film structure and mechanical response. Mechanical characterization of sub-100 nm films has been an ongoing challenge throughout the field and has gained tremendous interest with the growing prospects of organic electronics, which, at the device scale, possess an active layer of 100 nm or less^1–5^. To complicate matters, the properties of such nanometer-thin polymer films tend to deviate from their bulk properties due to an increasing contribution of the polymer interface^6,7^. This accompanies a phenomena known as the finite size effect, which describes the geometrical constraints that occur when the thickness of a polymer film is of the same length scale as the dimensions of an individual polymer coil, characterized by its polymer chain end-to-end distance (*R*_ee_)^8,9^. A prime example of such a thickness-dependent property is the glass transition phenomena in polystyrene (PS)^10–13^. For instance, reductions in the glass transition temperature (*T*_g_) as large as 10 and 80 °C have been reported for supported and free-standing (FS) 30 nm PS films^10,14^. More recently, the reduction in *T*_g_ with film thickness has been correlated to a decrease in film elastic modulus (*E*), which has not only invigorated interest in thin-film mechanics but also fundamental investigations surrounding *T*_g_ phenomena^15–18^.

Nanoindentation^19,20^ and buckling techniques^15,21^ have been used to measure the mechanical properties of ultrathin films deposited on silicon wafer or poly(dimethylsiloxane), respectively. However, the intrinsic properties of the film may be obscured by the underlying substrate due to sample–substrate interactions and a low signal-to-noise ratio. More recently, the pseudo-FS tensile test, referred to as film-on-water tensile test (FOW), has been used extensively in the study of both conventional and conjugated polymers (CPs). The FOW technique utilizes water as a nearly frictionless thin-film support, allowing the acquisition of complete stress–strain profiles^17,18,22–24^. However, there remains some concerns that the water support may influence such measurements, for instance, water acting as a plasticizer. Hence, there is a need for mechanical characterization without the supporting substrate, while maintaining the ability to analyze a wide range of polymer films with stiff (glassy) and soft (rubbery) characteristics.

Currently, there are four primary methods for the mechanical characterization of ultrathin (sub-100 nm) FS films. These include nanobubble inflation^25–27^, camphor-enabled transfer^28^, tensile tester for ultrathin FS films (TUFFs)^29^ technique, and a more recent guide frame technique^30^. Nanobubble inflation is performed by placing a thin film across an etched silicon nitride substrate with defined holes. Applied pressure forms nanobubbles, whereas atomic force microscopy (AFM) monitors the thermoviscoelastic response of the film. Creep compliance as a function of time and temperature has been measured for films as thin as 3 nm (polycarbonate), revealing reductions in *T*_g_ by over 120 °C^31^. The main drawback for nanobubble inflation is that the length scale of the measurement (micrometer hole diameter) prevents observation of large-scale deformation, which is of particular interest within the growing field of deformable electronics. Camphor-enabled transfer is a uniaxial tensile test, whereby a composite film is fabricated utilizing camphor as a sacrificial substrate. At elevated temperatures, the camphor sublimes, leaving the intact thin film behind. This technique has primarily been used to study the mechanics of graphene but polycarbonate films as thin as 100 nm have been successfully measured. The TUFF method is an extension of the FOW tensile test, whereby a film floating on water is connected to a support frame and translated vertically into air. The film is subsequently laser etched into a dog-bone geometry and mechanical properties measured using uniaxial tensile testing. Currently, moduli of ~30 nm PS films can be measured by this technique. The guide frame technique relies on transfer of the film to a wax paper substrate, evaporation of water, followed by pick up with a polyethylene terephthalate guide frame with supports. The frame is then connected to a tensile stage and the supports are melted prior to testing. This technique was utilized to study PS films with thickness ranging from 45 to 4319 nm and a wide variety of gauge dimensions, thus providing insight in how film geometries alter the apparent mechanical properties observed. The advantages and disadvantages of each measurement technique are summarized in Supplementary Table S1.

**Fig. 1:**
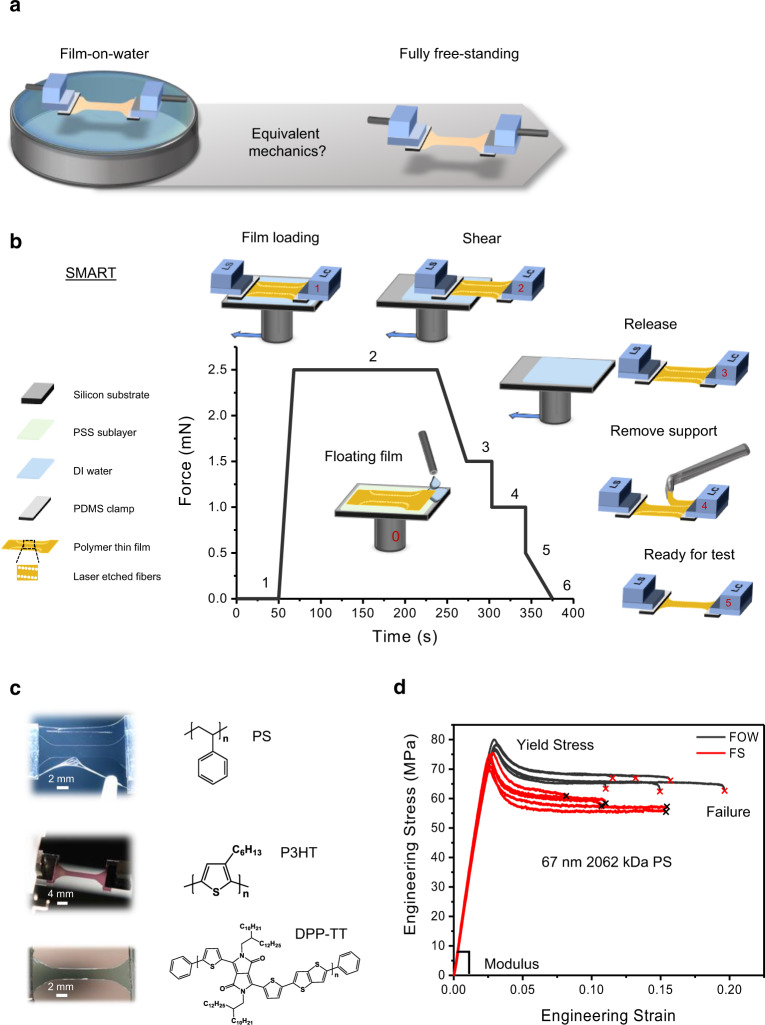
Use of shear motion-assisted robust transfer (SMART) method to obtain ultrathin films. **a** Illustration of the FOW and FS tensile tests. **b** SMART process and representative force applied to the film for obtaining a free-standing dog bone for tensile analysis. **c** The three polymers investigated within this study and representative free-standing films with a dog-bone gauge of 8 × 2 mm. From top–down: support removal from 19 nm high-molecular-weight PS, 80 nm P3HT prior to strain, DPP-TT prior to strain. **d** Representative stress–strain profiles for a 67 nm 2062 kDa PS film from both FS and FOW techniques.

Here we present an innovative shear motion-assisted robust transfer (SMART) process as a reliable technique to measure FS mechanics for both stiff glassy and soft viscoelastic polymers. We then compare the influence of water and air interfaces on the mechanics of ultrathin films (Fig. 1a) characterized by both FOW and FS techniques. The mechanical properties of three FS polymers, PS, poly(3-hexylthiophene) (P3HT), and 5-dialkyl-3,6-di(thiophen-2-yl)-2,5-dihydro-pyrrolo[3,4-c]pyrrole-1,4-dione (DPP)-containing polymer incorporating a linear thieno[3,2-b]thiophene donor (PDPP-TT), were measured and compared to results from the FOW test, to assess the influence of water. We observed minor differences in modulus, whereas yield stress (maximum strength) and strain at failure were slightly elevated for the FOW method, which is attributed to a force-dissipating mechanism provided by the capillary forces of the water. Independent measurements using quartz crystal microbalance (QCM) and neutron reflectometry (NR) demonstrated water uptake within PS and P3HT films to be as high as 9.79% and 9.13% by volume, respectively. This uptake in water supports that the mechanical properties of these hydrophobic polymer films are influenced by water to a minor extent, validating previous mechanical analysis using the FOW technique.

## Results

### SMART to obtain free-standing ultrathin films

The making of FS films for tensile measurements usually involves the transfer from a supporting substrate (silicon, glass) to the tensile stage. This is difficult, because the films are fragile and cannot easily be lifted off the supporting substrate. This remains true even for films supported by a water surface. If a 2 × 8 mm rectangular polymer film undergoes a vertical lift, the surface tension of water (~73 mN/m) results in an approximate downward/inward force of 1.5 mN, primarily along the perimeter of the film, causing local stress, bending, fracture, and ultimately rupture of the film. This effect is exacerbated in thin or brittle films, which have a tendency to fracture as seen with 60 nm PS (Supplementary Movie S1) and 300 nm graphene oxide films^28^.

Figure 1b and Supplementary Movie S2 demonstrate the SMART process used to obtain FS films. This approach is simple and includes the following steps. A laser-etched composite film (polymer of interest with outer microfiber support and poly(sodium 4-styrenesulfonate) (PSS) water-soluble sacrificial layer and silicon substrate) is attached to a motorized stage, which provides precise continuous shear speeds along the in-plane direction. Water droplets are placed at the corners of the silicon substrate, which then propagate throughout and dissolve the PSS sacrificial layer lifting the polymer thin film to a floating position. A linear stage and load cell are then attached to opposite ends of the film at which time a shear of 0.15 mm/s is applied to the silicon substrate parallel to the lateral dimension of the film, which is held stationary. At this speed, minimal lag exists between the water layer and the silicon substrate, which allows the removal of water from under the film while only forming a small mobile meniscus, which exerts minimal force on the film. Water–film contact angle and, subsequently, the force exerted on the film can be further reduced by adding ethanol to the water solution. Once an FS film is obtained, the laser-etched microfiber supports (example optical images are shown in Supplementary Figs. S1 and S2) are removed prior to mechanical analysis of the dog-bone-patterned film. The temporary supports help minimize wrinkling near the edges of the film as shown in supporting Supplementary Movie S2.

The force exerted on the polymer thin film was monitored throughout the SMART process by the attached load cell and is generally <3 mN. The shear process distributes this force more uniformly across the film and utilizing sacrificial side supports maintains the geometry of the dog bone. Upon removal of the side supports, an ~0.5 mN of force is estimated to have been applied to the final dog-bone-shaped film. A restoring force is then applied to the film, to remove any residual stress prior to measurement. For PS films, we demonstrate the potential of this technique to transfer films as thin as 19 nm (Supplementary Movie S3). Successful measurement of the thinnest films is further dependent upon removing defects, which may initiate failure during transfer or tensile testing. A facile method to mitigate such defects is to shorten the gauge length, thereby reducing the available area for defects during film formation and the length of travel during the SMART process. Dog bones with a gauge length of 4 mm (Supplementary Movie S3), as compared to longer gauge length of 8 mm (Supplementary Movie S2), were shown to provide a more efficient transfer, thus enabling increased data fidelity and successful transfer of the thinnest 19 nm films (success rate ~80%).

Here we demonstrate successful transfer and measurement of mechanical properties for a broad range of polymer films, including PS films (as thin as 19 ± 1.5 nm) as well as the viscoelastic CPs P3HT and PDPP-TT, as thin as 80 ± 3 and 75 ± 2 nm, respectively (Fig. 1c). Thus, this method can be applied to a broad range of thin-film materials.

### FS thin-film mechanics

Here we discuss the thin-film mechanics of rigid glassy and soft viscoelastic polymers, focusing on confinement and interfacial effects. PS, a glassy polymer with high *T*_g_, possesses fundamental interest, as it has been reported that sub-100 nm FS PS films, in excess of 350 kDa, exhibit a sharp reduction in *T*_g_, the severity of which increases with molecular weight. Sub-350 kDa PS features a more gradual *T*_g_ decay as seen with substrate-supported measurements^13,32,33^. The molecular weight-dependent *T*_g_ for FS PS is consistent with the finite size effect. In contrast, PS films with silicon support are independent of molecular weight and thus reductions in the *T*_g_ with reducing thickness more accurately represent the influence of the free surface mobile layer. The origins of the molecular-weight dependence for FS *T*_g_ are not yet understood and attracts significant attention^34^. In this report, we first explore the effect of PS molecular weight (183 and 2062 kDa) and gauge length (8 mm and 4 mm) on thin-film mechanics, using both FOW and FS techniques. Figure 1d shows representative stress–strain profiles for a 67 nm film measured with both techniques in the 4 mm gauge length dog-bone sample geometry. Particular attention is paid to 2062 kDa PS in hopes of observing mechanical properties with increased sensitivity towards the sample interface (air and water) given the range of viable thicknesses below the polymer’s large *R*_ee_ of ~94 nm.

**Fig. 2:**
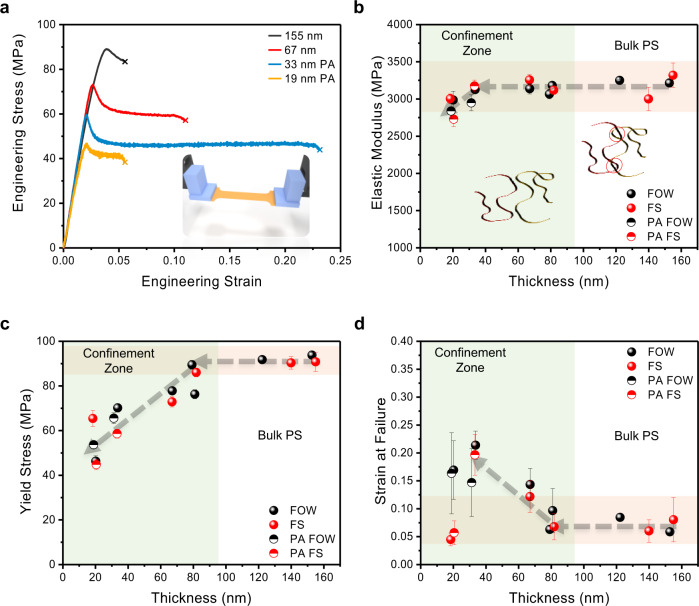
Comparison of 2062 kDa PS FS and FOW mechanical properties with reducing film thickness. Gauge length is 4 mm. **a** Representative stress vs. strain profiles of FS PS from 155 to 19 nm. **b** Elastic modulus thickness dependence of both FS and FOW PS films. Insert represents the loss of inter-entanglements upon confinement at thicknesses below the end-to-end distance of a polymer chain. **c** Yield stress thickness dependence of both FS and FOW PS films. **d** Strain at failure thickness dependence of both FS and FOW PS films. “PA” corresponds to samples characterized post vacuum annealing at 115 °C for 1 h. Error bars represent the SD of the characterized mechanical properties.

Tensile tests of 2062 kDa PS were performed for a series of films with thickness from 155 to 19 nm, exhibiting yielding and plastic deformation behavior (Fig. 2a and Supplementary Figs. S3–S5). Large-scale deformation of a 19 nm-thick PS film was monitored by optical microscopy, demonstrating wrinkling and then shear deformation zones post yielding (Supplementary Movie S3 and Supplementary Fig. S6). To the best of our knowledge, this measurement is the thinnest FS PS film to undergo tensile testing, an ~40% reduction in thickness from the previous record of 30 nm^29^.

Regardless of the molecular weight, a near-equivalent modulus was observed between the FS and FOW techniques at all film thicknesses (Fig. 2b and Supplementary Figs. S3b and S7a), indicating that polymer–water and polymer–air interfaces have similar effects on the thin film’s mechanical properties. This observation is consistent with measurements of PS nanoparticles in aqueous solution, which have demonstrated equivalent *T*_g_ reductions to those of sub-350 kDa FS films^35^. Both techniques, in the 8 mm-gauge length dog-bone sample geometry, demonstrate a reduction in modulus lower than the bulk value for sub-40 nm thin films, consistent with a previous study using buckling metrology^15^.

Films were subjected to vacuum thermal annealing at 115 °C for 1 h to eliminate residual stress. Post annealing, the modulus of 8 mm sub-40 nm PS films increased to the bulk value of ~3 GPa, excluding FS 19 nm PS, which remained just under the bulk *E*. This increase in modulus upon annealing indicates that the previously observed reduction originates from a process-driven phenomenon, unrelated to *T*_g_^36^. We attribute this effect to residual stress from rapid evaporation of solvent during spin casting, which results in radially aligned polymer chains, thus reducing the number of load-bearing chains in the tensile direction and lowering the apparent *E*^37,38^. Upon annealing above the bulk *T*_g_, the polymer chains relax isotropically, leading to a reduced orientation and an increased number of load-bearing chains. PS films exceeding 40 nm in thickness, excluding 153 nm, were not annealed as the bulk modulus was maintained. This implies a critical thickness for PS films wherein residual stress begins to influence the apparent *E*. Previous mechanical analysis of a cast ~220 nm PS film also demonstrated identical modulus to films annealed at 115 °C for 15 h^17^. We note that 4 mm thin films do not demonstrate a strong dependence on annealing, which may be due to the reduced size scale limiting the influence of anisotropy, and further investigation is warranted.

From a *T*_g_ perspective, the increase in *E*, post annealing, for 183 kDa PS is expected as at 38 nm in thickness, the anticipated *T*_g_ from literature is near 80 °C^39^, sufficiently above the measuring temperature of 25 °C, and thus should express bulk-like behavior. In contrast, the bulk modulus of the 19 nm 2062 kDa FS PS film is particularly interesting, given that at such high confinement (21% of *R*_ee_), the *T*_g_ is expected to lie below room temperature^13^ where the film would reside within the rubbery regime and modulus should exhibit a considerable reduction^40^. That is not the case here and thus points to an alternative *T*_g_ phenomenon.

Here we seek to explain why the modulus of the 19 nm FS PS film maintained its high value despite heavy confinement. Seminal work by Forrest et al.^41^ utilized Brillouin light scattering to investigate the high-frequency mechanics of FS 767 and 2240 kDa PS films as thin as 29 nm, which surprisingly demonstrated bulk-like properties. This finding is congruent with more recent *T*_g_ investigations of FS PS by Connie Roth where ellipsometry covering a wider temperature range revealed two *T*_g_’s^14,42^. The lower *T*_g_, ~20 °C for 30 nm films of 934 kDa PS, was found to be molecular weight dependent, consistent with previous *T*_g_ measurements of high-molecular-weight FS PS^39,43^, whereas the higher *T*_g_, ~85 °C, was observed to be molecular weight independent, consistent with both supported PS and sub-350 kDa FS PS films^13^. The high *T*_g_ fraction, independent of molecular weight, was determined to contribute up to 90% of the thermal expansion, making it the dominant transition. This observation is consistent with work by O’Connel et al.^27^ using nanobubble inflation, which demonstrated a similar trend in *T*_g_, ~80 °C and 60 °C for 994 kDa PS films with thicknesses of 30 and 20 nm, respectively, as well as an independence of molecular weight^27^. Considering these works, the measurement temperature (~25 °C) for our 19 nm FS PS films lies significantly within the glassy state and is thus congruent with our mechanical analysis, demonstrating high modulus and molecular weight independence. Furthermore, there is a discrepancy between the enhanced local dynamics observed with FS films and large-scale chain motion, which has been shown to express bulk-like behavior. McKenna and colleagues^25,27^ have reported rubbery stiffening, as well as glassy stiffening, of thin PS films at reduced thicknesses, despite reductions in the thin film *T*_g_. This indicates that long-range and local chain dynamics respond differently when confined, and may potentially obfuscate the expected mechanical response from *T*_g_ alone. Hole formation, an indicator of large-scale chain mobility, has also been shown to occur near the bulk *T*_g_ for FS PS films, regardless of the apparent *T*_g_ reduction occurring in such thin films^44,45^. Considering that tensile deformation also represents large-scale chain motion, it is possible that FS films experiencing tensile deformation, as within this work, may similarly be negligibly affected by the apparent thin film *T*_g_ as in hole formation. Ediger et al.^7^ measured the molecular motion of FS PS films, >14 nm thick, which were seen to possess a bulk-like interior with a maximum mobile layer thickness of 7 nm at elevated temperatures near the bulk *T*_g_. Experiments performed below ~80 °C demonstrated a mobile layer thickness of <1 nm. This finding was conjectured to support the resistance of thin films toward hole formation^46^. In regards to the current study, this suggests that the mobile layer contributes <5.3% of the polymer volume for a 19 nm thin film and thus would minimally impact modulus in most instances. Although bulk-like modulus is observed, we note a 10% average reduction in modulus for confined 19 nm films relative to the unconfined state (Fig. 2b), congruent with the above discussion. Previously, a reduction of modulus for 136.5 kDa PS was observed near a thickness of 25 nm and was correlated to the *R*_ee_ of 25 nm^17^. As the reduction in modulus in this work also occurs near this thickness despite a greater *R*_ee_ of 94 nm, we hypothesize that such reduction is due to the mobile interface and not geometric constraints associated with confinement below the *R*_ee_. Lastly, the bulk modulus observed in this report agrees with previous results from FOW and TUFF measurements, despite heavy confinement^23,29^. Thus, we conclude that the thickness-dependent modulus values seen in FS PS films are (1) independent of molecular weight, (2) independent of conformational restrictions associated with confinement below the *R*_ee_ of a polymer chain, and (3) primarily dictated by the polymer–surface interface, as is the case for supported thin-film *T*_g_ measurements.

Beyond the modulus of 2062 kDa PS, a near-linear reduction in yield stress was observed throughout the confined films regardless of the technique employed (Fig. 2c and Supplementary Fig. S3c). For the 8 mm films, the yield stress and strain at failure were reduced relative to the FOW technique (Supplementary Figs. S3 and S4). We believe this is due to potential defects along the transferred films, which upon reducing the gauge to 4 mm was mitigated (Supplementary Fig. S5). For 183 kDa PS, average strain at failure was observed to be greater in the FOW measurements, whereas yield stress did not show a strong trend (Supplementary Figs. S7 and S8). It is important to note that although modulus does not correlate to the polymer *R*_ee,_ yield stress and strain at failure are marked by a clear transition to lower and higher values, respectively, upon confinement (Fig. 2d). The 19 nm films demonstrate a difference of 43% in the yield stress and the 30 nm films demonstrate a 160% difference in strain at failure relative to the unconfined films. In addition, confined FOW measurements have an average increase in yield stress and strain at failure by 4.3% and 3%, respectively. The difference in strain at failure does not include the 19 nm films, which show disagreement between the two techniques. This difference may be associated with a loss of inter-entanglements resulting in the reduced strain at failure for the FS film, whereas in the case of FOW measurements this is mitigated by the water interface. We note significant wrinkle formation within the FS films under tensile strain relative to the FOW measurements (Supplementary Fig. S9). This may indicate that the surface tension of water is sufficient to retard strain localization in thin films resulting in a more uniform deformation, similar to films stretched on an elastomeric substrate, and thus deter crack propagation. Although small, these results support the concept of a force-dissipating mechanism provided by the water interface, resulting in elevated yield stress and strain at failure for confined films. This makes conceptual sense as the polymer–water interface begins to play a greater role at reduced thicknesses but may be overcome by the loss of inter-entanglements when significantly confined. Both the trend in modulus and strain at failure are in agreement with that reported by Bay and Crosby^29^ using their TUFF technique. However, we note that a distinguishing feature between these data sets is the low strain at failure exhibited from the TUFF technique, thereby limiting the observable deformation of 151.5 kDa PS to the onset of the yielding regime (<2.5% strain), whereas in the SMART process strain at failure upwards of 15% strain (85 nm 183 kDa) is observed, surpassing the yielding point and fully within the plastic deformation zone. Considering that these techniques and the PS materials used are fundamentally similar, it is important to ascertain the cause for such variations in ductility. We propose two potential origins for the low ductility found through the TUFF technique. (1) PS films were annealed at 170 °C for 25 min, which may result in hole formation as previously shown for 71 nm PS on Krytox oil after annealing at 160 °C^47^. To further explore this possibility, we annealed ~40 nm PS on both mica and silicon substrates at 170 °C for 25 min, resulting in partial de-wetting of the PS film (Supplementary Fig. S10). (2) Previous measurements by our group using the FOW technique have shown the strain at failure for PS to decrease at elevated strain rates^18^. Considering that the strain rate used through the TUFF methodology is 16 times greater than the strain rate of 5 × 10^−4^ s^−1^ used in the current study, this may be a significant causative factor in the relatively low strain at failure. Regardless, the enhanced ductility from the FOW measurements may be due to a force-dissipating mechanism provided by the water interface. If this is the case, then it is expected that similar phenomena may occur in other hydrophobic polymer thin films.

**Fig. 3:**
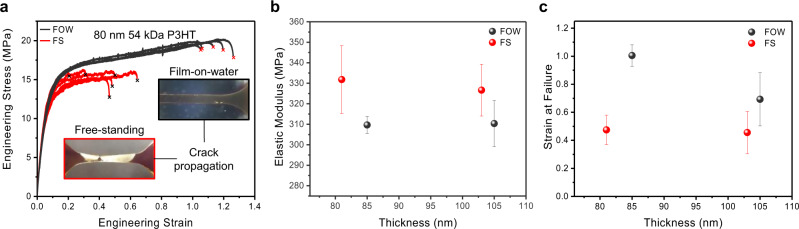
Comparison of 25 kDa P3HT FS and FOW mechanical properties at ~105 and ~80 nm using the 8 mm-gauge geometry. **a** Representative stress vs. strain profiles of 80 nm P3HT for both FOW and FS tensile test. Insert is an optical comparison of a FS and FOW P3HT at failure. **b** Modulus and **c** strain at failure of P3HT in both FOW and FS measurements. Error bars represent the SD of the characterized mechanical properties.

Mechanical analysis was performed on a more challenging ductile polymer, P3HT, the benchmark CP. P3HT is a soft viscoelastic polymer, which has a bulk *T*_g_ near room temperature^48^ and, consequently, a relatively low modulus of 100–350 MPa, depending on measurement technique, sample crystallinity, regioregularity, and molecular weight^18,49–51^. As such, this polymer represents a significant challenge, as the forces applied during any transfer process may lead to irreversible deformation and thus alter the measured mechanical properties. In this study, we investigated the mechanics of 105- and 80 nm-thick P3HT films, which, to our knowledge, is the first sub-100 nm FS mechanical analysis for any CP reported to date. All measurements were performed without annealing given that the sub-room temperature *T*_g_ of P3HT should limit anisotropy from spin coating. Figure 3a and Supplementary Fig. S11a show the stress–strain profiles for 80 and 105 nm films, respectively, under both FOW and FS techniques. From these curves, it is readily apparent that the yield stress and strain at failure are lower for the FS measurement, whereas the difference in modulus is less noticeable with a value ~6% greater (Fig. 3b, c). These values track the trends seen in the PS films, although more significantly, and support the notion of water providing a mitigating mechanism towards crack propagation. As stated previously, it is possible given the viscoelastic characteristics of P3HT, that some form of plastic deformation throughout the SMART process may lead to a reduction in yield stress or strain at failure. This is not believed to be the case given that a significant reduction in apparent modulus would also be expected, whereas we note a ~6% increase in FS compared to FOW, from 309 to 332 MPa and from 310 to 326 MPa for 80 and 105 nm, respectively. However, this difference could imply a slight plasticization effect from the water, which may explain the decreasing differences in mechanical properties at the greater thickness of 105 nm (i.e., lower Δ *modulus*, Δ strain at failure, and Δ yield stress between FS and FOW). Considering the hydrophobic nature of P3HT, this would seem unlikely at first glance^52^. Thus, we will also discuss the diffusion of water into such polymer films in a later section.

Preliminary mechanics of PDPP-TT, a high-performance donor–acceptor (DA) CP, was also investigated at 75 nm in thickness for both methods (Supplementary Fig. S11b). PDPP-TT possesses a variety of chemical functional groups (Fig. 1c), different than PS or P3HT, which may lead to altered mechanical performance depending on interactions at the interface. Of most concern is the carbonyl group, which may form hydrogen bonds with water and potentially plasticize the film. However, similar modulus was observed between the two techniques and strain at failure was found to be slightly greater in the FOW measurements. Further investigation is necessary to confirm this, but preliminary results parallel our previous analysis on PS and P3HT polymers, where modulus was observed to change less significantly than strain at failure or yield stress. Thus, both methods are viable, in particular for modulus measurement, for DA polymers, which often possess carbonyl functionalities. However, this is not to suggest that this would be the case regardless of composition, as the side-chain content and concentration of such functional groups differ considerably among synthesized CPs, such as hydrophilic functionality for bioelectronic applications.

### Characterizing the presence of water

**Fig. 4:**
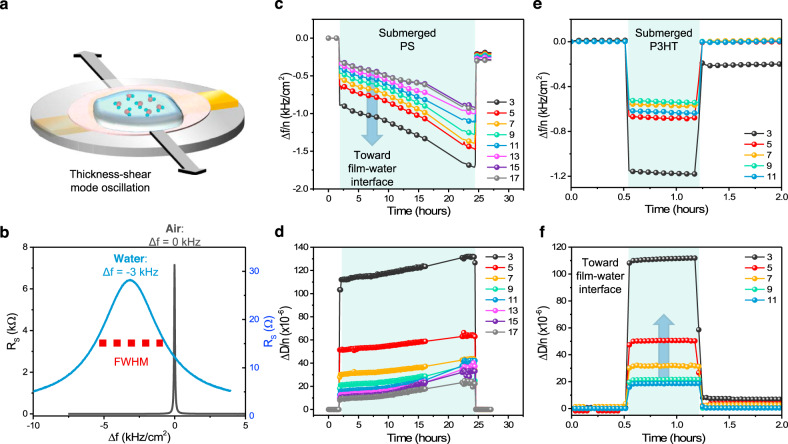
Quartz crystal microbalance analysis of PS and P3HT films submerged in DI water. **a** Illustration of film (pink) on the quartz sensor submerged in water. **b** Representative response of QCM coated with P3HT during transfer from air to water. FWHM corresponds to full width at half the maximum intensity. **c**, **d** Normalized frequency shift and change in energy dissipation of ~100 nm PS submerged in water for 20+ h. **e**, **f** Normalized frequency shift and change in energy dissipation of ~100 nm P3HT submerged in water for ~40 min.

To ascertain the presence of water in these hydrophobic films (PS and P3HT), we utilized QCM and NR. QCM is a technique that can provide a qualitative understanding of both mass uptake and energy dissipation (change in stiffness) throughout the depth of a film with particular sensitivity towards the interface^53,54^. This is accomplished through tracking the response of multiple crystal harmonics (*n*), whereby low *n* harmonics correspond to regions closer to the film–water interface of interest and higher *n* harmonics probe the film–substrate interface. The QCM measurement was conducted with each film submerged in water, using the initial air response as a reference (Fig. 4a, b). Figure 4c shows an immediate reduction in the normalized frequency shift (Δ*f*/*n*) of 100 nm PS upon submersion. This shift corresponds to a gain in mass from the water, which primarily occurs at the film–water interface, given that the lowest harmonic *n* = 3 has the largest frequency shift. Furthermore, the full-width half max, which is a measure of the normalized energy dissipation (Δ*D*/*n*), was found to increase towards the film–water interface, signaling a softening or plasticization effect from the water (Fig. 4d). Throughout the experiment, there was a slow gain in mass and continuous softening of the film throughout its thickness. Upon drying, Δ*D*/*n* returns to the initial reference value, indicative of the reversibility of the transition. P3HT (100 nm) shows a rapid response and stabilization in water (Fig. 4e, f). Most significant mass gain and softening occur near the film–water interface (indicated by lower *n*), suggesting a diffusion-limited process. Response is not reversible at low harmonics (third harmonic trace does not return to Δ*f*/*n* = 0 after removal from water), suggesting possible morphological or structural change during the interaction with water. QCM results confirm the presence of water within the bulk PS and P3HT films, and indicate that water softens these hydrophobic films to some extent and does so primarily at the film–water interface. This may explain the 6% difference in modulus observed for P3HT FS and FOW measurements. In contrast, the value of modulus of PS thin film is relatively stable, even when strongly confined, and thus we consider the following possibilities: (1) water may be penetrating through the film via pinhole defects originating through sample preparation. Given a small enough number of defects, the mechanical properties may not be influenced, despite detection by QCM. (2) The softening effect shown by QCM may be small and, thus, not influence the large modulus of PS. For example, an approximate reduction of 20 MPa was observed for the modulus of P3HT, but for PS this same reduction would be indistinguishable given the typical modulus of 3 GPa and uncertainty of ~100 MPa. Thus, glassy hydrophobic thin films with high modulus may be less influenced by diffusion of water. To investigate further, 90 and 70 nm PS films, 183 and 2062 kDa, respectively, were exposed to water for 48 h prior to tensile measurement. Modulus and yield stress did not deviate significantly from the standard measurement, whereas strain at failure significantly increased in both cases (Supplementary Fig. S12). Although enhanced ductility is often a side effect of plasticization, the small influence on modulus and yield stress suggests some other mechanism may be at work. This coincides well with the QCM response at 24 h, where water uptake doubles but the energy dissipation (softening) remains relatively stable compared to the initial response upon submersion. Thus, the increase in water content at extended times and the subsequently greater ductility indicates that water mitigates crack propagation. In this respect, we chose to further explore NR, which has high sensitivity with nanometer resolution in the film thickness direction, to quantify the uptake of water.

**Fig. 5:**
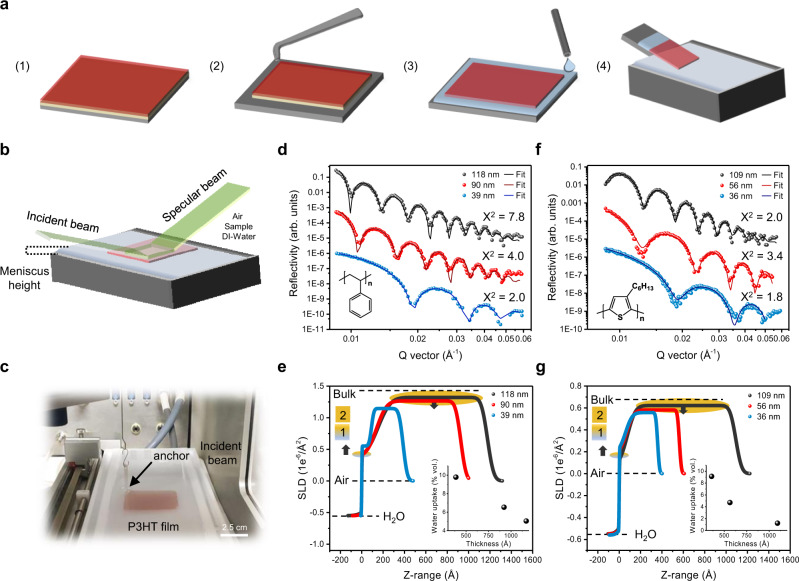
Monitoring water uptake within PS and P3HT thin films via neutron liquid reflectometry. **a** Sample loading with (1) spun cast composite film with PSS underlayer, (2) removal of film edge, (3) floating of film, (4) transfer into trough. **b** Illustration of NR experiment with film floating on the water surface. **c** Photograph of a 5 × 5 cm P3HT film of 36 nm in thickness anchored within the DI-water trough. **d** Reflectivity vs. wave vector for PS films of varying thickness. **e** PS SLD profile in *Z*-direction. Insert is of PS water uptake dependence on thickness. **f** Reflectivity vs. wave vector for P3HT films of varying thickness. **g** P3HT SLD profile in *Z*-direction. Insert is of P3HT water uptake dependence on thickness. The shaded regions corresponding to the sublayer and top layer represent the layers 1 and 2 depicted in Fig. 6.

To more accurately match the environmental conditions of our FOW tensile test, NR measurements were performed at room temperature with a 5 × 5 cm film floated onto a water trough, thus mimicking the water–sample and sample–air interfaces present in the FOW test (Fig. 5a–c). Figure 5d shows the NR results and best fits for PS with thickness ranging from 118 to 39 nm. Each film was interpreted using a two-layer model: layer 1 adjacent to the water interface (sublayer) and layer 2 adjacent to the air interface (top layer). This two-layer model was supported by the previous QCM analysis, which demonstrated the film–water interface to have the most significant response, while higher *n* harmonics revealed a more gradual response. The thickness, scattering length density (SLD), and roughness of the layers were systematically varied and optimized until the sum of the *χ*^2^-values for all the data points were minimized (Supplementary Table S2 for fitting results)^55^. The thickness of the films were determined by *h* = 2*π*/Δ*Q*, where Δ*Q* is the wave vector difference between fringes. The obtained SLD profile is provided in Fig. 5e and directly describes the water uptake within the films given the following equation:

1 $${\mathrm{SLD}}_{{\mathrm{film}}} = {\mathrm{SLD}}_{{\mathrm{PS}}} \ast \left( {1 - {{x}}} \right) + {\mathrm{SLD}}_{{\mathrm{H}}2{\mathrm{O}}} \ast {{x}}$$

where *x* represents the volume fraction of water within the film and the calculated values for SLD_PS_ and SLD_H2O_ are 1.42 × 10^−6^ and −0.56 × 10^−6^ Å^−2^, respectively. Reflectivity data of the dry films (Supplementary Fig. S13) demonstrated a reduction in SLD with thickness from 1.42 × 10^−6^ to 1.33 × 10^−6^ Å^−2^, which was attributed to the growing contribution of the mobile interface. We observe a reduction in the SLD_film_ with decreasing thickness, from 1.321 × 10^−6^ to 1.146 × 10^−6^ Å^−2^ for the 118 and 39 nm films, respectively. These values correspond to an increase in the volume fraction of water, present in the top layer, from 5.04% to 9.79%, a significant amount given the hydrophobicity of PS. This is in contrast to previous work on deuterated PS where no water uptake was observed^56^. The primary difference between these measurements and those reported by Tanaka and colleagues^56^ are the environmental conditions at the interface of the polymer. The previous work was performed with deuterated PS supported by quartz substrate, meaning that there is both an air–polymer and polymer–quartz interface. In the current work, the PS film is directly floated on water where two mobile interfaces exist, air–polymer and polymer–water. This may facilitate water uptake given the potential for enhanced dynamics exhibited by both interfaces. In addition, the uptake in water for PS is also supported by the previously discussed QCM results. The water–film interface or sublayer is more complex, as an increase in SLD is observed (0.361 to 0.552 × 10^−6^ Å^−2^) with reducing thickness. We attribute this to a reduction in roughness with decreasing film thickness (Supplementary Table S2), as greater roughness will raise the apparent water concentration across the film interface leading to the lower SLD. Time-dependent measurements were also conducted with the same PS films after a 4 h exposure to water. No significant changes were observed in the thicknesses of the films and SLD_film_ exhibited a marginal reduction (Supplementary Fig. S14). This stability supports the QCM findings, indicating that the diffusion of water into PS films is relatively slow after the initial exposure to the surface.

**Fig. 6:**
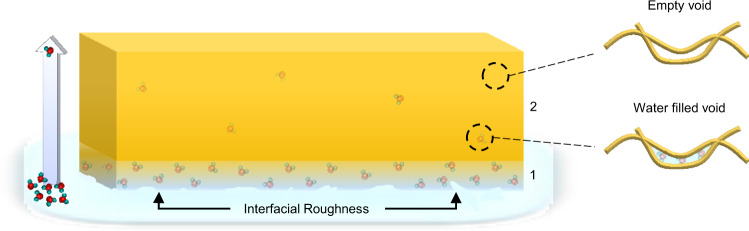
Illustration of water residing within a representative hydrophobic film, with the majority of water present at the relatively rough polymer–water interface. Layer 1 depicts decreasing water content with increasing distance from the film–water interface followed by a reduced water content in layer 2. “Wet” and “dry” polymer chains are depicted on the right, illustrating that the water resides within pre-existing voids throughout the film and does not swell the polymer chains.

Similarly, P3HT was also studied using NR with film thicknesses from 109 to 36 nm (Fig. 5f, g). The results are comparable to those of PS. We observe a reduction of the SLD_film_ from 0.622 to 0.558 × 10^−6^ Å^−2^ with decreasing thickness, which corresponds to water volume fractions of 1.24% and 9.13%, respectively. Thus, from QCM and NR, we were able to confirm that water does penetrate these hydrophobic films, and that plasticization and its effect on modulus are relatively small at the film thicknesses used in our mechanical measurements. This fact is illustrated in Fig. 6, where water primarily resides near the rough film–water interface and also diffuses throughout the film with a decreasing gradient. We hypothesize that the water primarily lies within voids throughout the film but does not significantly swell adjacent to polymer chains, given the slight influence on modulus as previously observed. However, the presence of water within these films, especially the increased volume fraction observed at low thicknesses, supports the claim that water is responsible for the elevated strain at failure and yield stress found throughout our FOW tensile measurements.

In summary, we have introduced the SMART method to measure FS thin-film mechanics. First, the influence of confinement on FS PS thin films was explored. Despite heavy confinement, only the thinnest FS PS film of 19 nm demonstrated a reduction in modulus (10%). Our results indicate that film modulus is dependent on a mobile interface rather than on geometric confinement below *R*_ee_. This is in contrast with previous reports of high-molecular -weight 20 nm FS PS films exhibiting sub-room temperature *T*_g_. However, yield stress and strain at failure show a transition to lower and higher values, respectively, when confined below the *R*_ee_. Second, the difference between FS and FOW mechanics was then explored for three polymer systems (PS, P3HT, and PDPP-TT) representing both stiff glassy and soft viscoelastic materials. Modulus and yield stress did not differ considerably between the two measurements, whereas strain at failure were consistently greater for the FOW measurements. This difference increased with both softer polymer films (P3HT) and increased water exposure time (PS). Despite the hydrophobic nature of these films, water content as high as 9.79% by volume was observed with a primary contribution occurring at the film–water interface. Upon tensile strain, the water interface provides force delocalization resulting in elevated yield stress and strain at failure. This SMART technique provides a new means of studying the mechanics of thin films and two-dimensional (2D) materials inaccessible to existing techniques.

## Methods

### Materials

PS was obtained from Polymer Source with weight-average molecular weights/dispersity of 183 kDa/1.06 (low molecular weight) and 2062 kDa/2.9 (high molecular weight). The weight-average molecular weight for 2062 kDA was characterized by gel permeation chromatography using trichlorobenzene as the eluent at 160 °C, PS for calibration, viscometer, and light scattering as the detectors (Supplementary Fig. S15). P3HT and PSS were obtained from Sigma-Aldrich with weight-average molecular weights/dispersity of 54 kDa/2.74 and 70 kDa, respectively. PS was annealed under vacuum to remove solvent impurities, whereas the other samples were used as received without additional purification or GPC measurement. PDPP-TT was synthesized following previously established procedures^57^. The weight-average molecular weight was determined to be 184 kDa with a dispersity of 3.62 via characterization by GPC as described above for 2062 kDa PS.

### Dog-bone film preparation

The composite films were fabricated by first spin casting 3 wt% PSS in aqueous solution onto plasma-treated silicon wafer at 4000 r.p.m. for 1 min. The polymers of interest were then spun cast from their respective solutions at 2000 r.p.m. for 2 min. Concentrations are as follows: 183 kDa PS: 25, 20, 15, 10, and 7.5 mg/ml solution in toluene; 2062 kDa PS: 15, 13, 10, 7.5, 5, and 3.5 mg/ml solution in toluene; P3HT: 25 and 20 mg/mL solution in chlorobenzene; and DPP-TT: 10 mg/ml solution in chlorobenzene.

Unless stated within the discussion, all samples were measured as cast without annealing. Some samples were annealed in a vacuum oven at 115 °C for 1 h and allowed to naturally cool to room temperature while under vacuum.

Once the composite films were fabricated, they were then laser etched using a Ytterbium 20 W laser with a wavelength of 1064 nm. The dog-bone gauge length and width were etched to either 8 × 2 or 4 × 2 mm, respectively, with a 3.25 mm-wide support on each side of the gauge. Microfibers connecting the dog bone to the side supports were then laser etched. Samples were then separated into individual dog bones for testing.

### Thickness measurement and film uniformity

After creating the composite film, a representative portion was floated on water, removing the PSS layer, and collected on fresh silicon. Film thickness was determined using AFM for all polymer materials. In addition, a F20-UVX interferometer was used to measure the thickness of PS films, utilizing a probing wavelength from 325 to 1700 nm, refractive index of 1.5865, and a spot size of 1.5 mm in diameter.

Film uniformity is assessed in Supplementary Fig. S16. To assess the uniformity of the thinnest 19 nm film, a fresh 3.5 mg/ml solution of PS in toluene was spun cast onto a 4 × 4 cm wafer with PSS layer. The film was divided into nine 1 × 1 cm squares as demonstrated below, where each PS film was floated on water, removing the PSS layer, and collected on fresh silicon. Both AFM and interferometry were used to assess thickness throughout each coordinate. Three measurements were performed at different locations within each 1 × 1 cm coordinate. In addition, NR was performed on 19 nm PS film without PSS, verifying that spin coating on a 5 × 5 cm wafer provides a uniform thickness.

### FS tensile test

Each sample was connected to a motorized *x*-stage using a vacuum pen for easy sample handling. Deionized water droplets were then placed at each corner of the film, to dissolve the PSS underlayer and lift the film from the silicon surface. Once lifted, the linear stage and load cell poly(dimethylsiloxane) clamps were attached to the pads at each end of the dog-bone film. An approximate shear speed of 0.15 mm s^−1^ was then applied with the motorized *x*-stage, while monitoring the water meniscus across the film. In general, residual water on the film was minimal at this shear speed and resulted in a pristine FS film. A tweezer was then used to remove the outer film supports, which was facilitated by the laser-etched microfibers. The film was then left in air to dry and equilibrate prior to tensile testing. During the tensile test, each film was elongated with an applied strain rate of 5 × 10^−4^ s^−1^, while simultaneous measurement of the force was conducted at a frequency of 10 Hz. The force-displacement data were then converted into the representative stress–strain plots.

The pseudo-FS tensile test is similar to that reported here and is described in great detail in our previous publications^18,24^. However, in this report, laser etching was utilized as the primary means of dog-bone patterning rather than oxygen plasma etching. The laser-etching process provides equivalent mechanical properties to films prepared by oxygen plasma etching, as shown in Supplementary Fig. S17, despite differences in edge appearance.

The correction factor for the 4 mm dog bones was determined by dividing the modulus of the 8 mm films measured on water to that of the 4 mm films of similar thickness. An average correction of 1.314 with a SD of 0.056 was determined. For the thinnest films, only the annealed measurements were compared to discount the influence of processing conditions. This correction factor was then multiplied by the gauge length of 4 mm to yield an apparent gauge length of 5.256 mm. This apparent gauge length corrects the strain values of the 4 mm films and, in turn, provides an accurate comparison of modulus and strain at failure for the 8 mm films. Yield stress is not influenced by this correction but rather the strain at which yielding occurs.

### Quartz crystal microbalance

P3HT and PS films of 100 nm thickness were deposited on Au-coated 5 MHz Au-coated QCM crystals by spin coating at 2000 r.p.m. for 30 s. QCM measurements were carried out by tracking frequency shift and change in peak width of the first nine off-crystal harmonics using a SARK-110 vector impedance analyzer controlled using custom software written in Python. Gravimetric/viscoelastic response of films was measured, while the film was transferred from air and submerged in Milli-Q water, and then transferred back to air. Values of Δ*f* and Δ*D* were calculated by fitting QCM conductance peaks to a Lorentz distribution and extracting position and width^54,58^.

### Neutron reflectometry

NR measurements were performed on the Liquids Reflectometer (LIQREF), BL-4B, at the Spallation Neutron Source in Oak Ridge National Laboratory with a 2D position-sensitive ^3^He detector^59^. The reflected intensity was measured as a function of the momentum transfer *Q* = 4*π* sin*θ*/*λ*, where *θ* and *λ* are the incident angle and wavelength of the neutron beam, respectively. A 6.8 Å bandwidth, selected from a wavelength range of 5.95–15.68 Å, is used in conjunction with measurement angles of *θ* = 0.60, 0.75, 1.1, and 1.62 to span a *Q* range of 0.008–0.060 Å^−1^. The footprint of the beam was 35 × 20 mm^2^, smaller than the 50 × 50 mm^2^ dimension of the films, and was kept constant by increasing the beam-defining slit openings proportional to the neutron angle of incidence. PS or P3HT films were floated onto the meniscus of a DI-water Langmuir trough through the release of a PSS layer, identical to the process in the FS tensile test. A small scratch was made along each edge of the square film and DI-water droplets were placed at each corner to allow a gentle release from the silicon substrate as the PSS layer dissolved. Once fully lifted, the floated sample (still on silicon) was deposited into the Langmuir trough, which was then sealed to minimize air vibration and water evaporation. The entire system was supported by the anti-vibration table (Accurion Halcyonics, MD, USA). Each sample was exposed to the room-temperature water surface for 1 h during the alignment procedure and subsequently scanned with a *Q*-vector range from 0.008 to 0.060 Å^−1^. Data reduction and fitting were performed through the online reflectivity modeling software provided by BL-4B^55^.

## Supplementary information

Supplementary Information

Description of Additional Supplementary Files

Supplementary Movie 1

Supplementary Movie 2

Supplementary Movie 3

## Data Availability

All relevant data in this study are available from the corresponding author upon request.
