# Supplementary material for: SMART transfer method to directly compare the mechanical response of water-supported and free-standing ultrathin polymeric films
Source: Nat Commun. 2021 Apr 20;12:2347. doi: 10.1038/s41467-021-22473-w (PMC8058343; doi:10.1038/s41467-021-22473-w)
Supplement: Supplementary file 1 — Supplementary Information [file 41467_2021_22473_MOESM1_ESM.pdf]

Supplementary Information

SMART Transfer Method to Directly Compare the Mechanical Response of Water-Supported and Free-Standing Ultrathin Polymeric Films.

*Luke A. Galuska[1], Eric S. Muckley[2], Zhiqiang Cao[1], Dakota F. Ehlenberg[1], Zhiyuan Qian[1], Song Zhang[1], Simon Rondeau-Gagné[3], Minh D. Phan[4], John F. Ankner[4], Ilia N. Ivanov[2], Xiaodan Gu[1]**

[1]Center for Optoelectronic Materials and Devices, School of Polymer Science and Engineering, University of Southern Mississippi, Hattiesburg, MS 39406, USA

[2]Center for Nanophase Materials Sciences, Oak Ridge National Laboratory, Oak Ridge, TN 37830, USA

[3]Department of Chemistry and Biochemistry, University of Windsor Ontario N9B3P4, Canada

[4]Neutron Scattering Division, Oak Ridge National Laboratory, Oak Ridge, TN 37830, USA

*Email: xiaodan.gu@usm.edu

Supplemental Figures

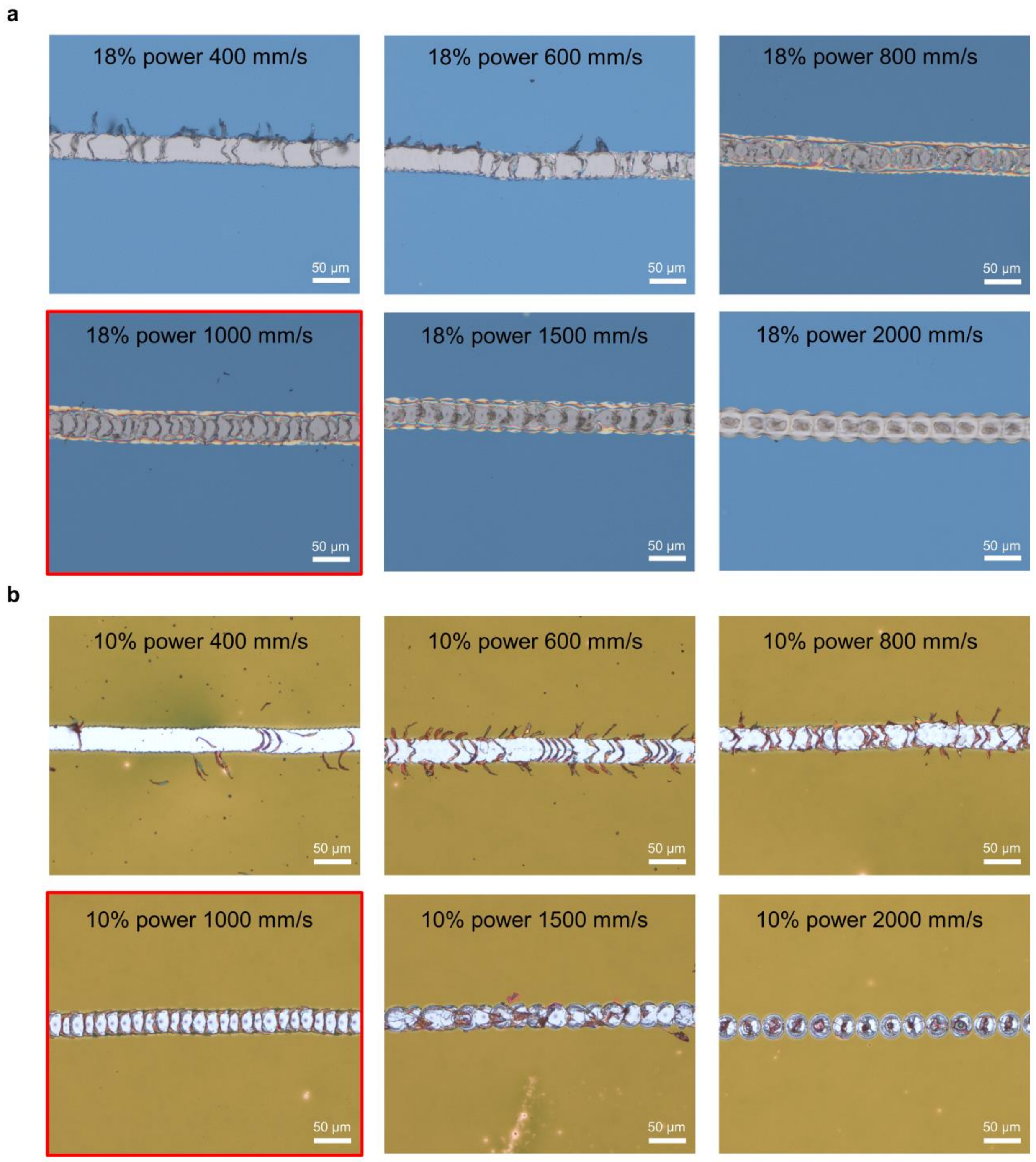

Figure S1: Optical images of a) 80 nm PS and b) 80 nm P3HT laser etched under different parameters to create the desired microfiber support. Outlined in red are the desired laser etched fibers for sufficient support throughout the transfer process but weak enough for smooth removal.

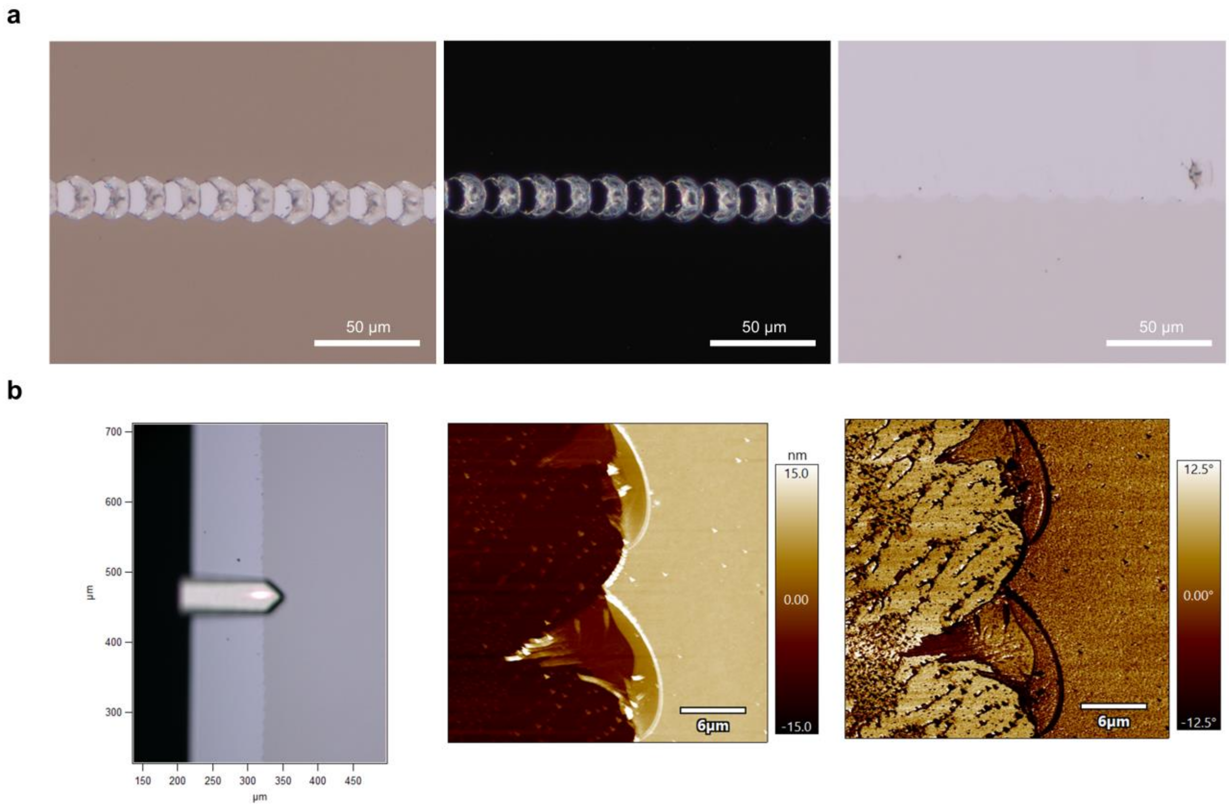

Figure S2: Evaluation of 19 nm film integrity before and after side support removal. a) Optical microscopy images of laser etched (4% power) 19 nm PS prior to removal (bright field and dark field) and after removal (right). b) Atomic force microscopy of film edge after removal of side support (optical, height, phase).

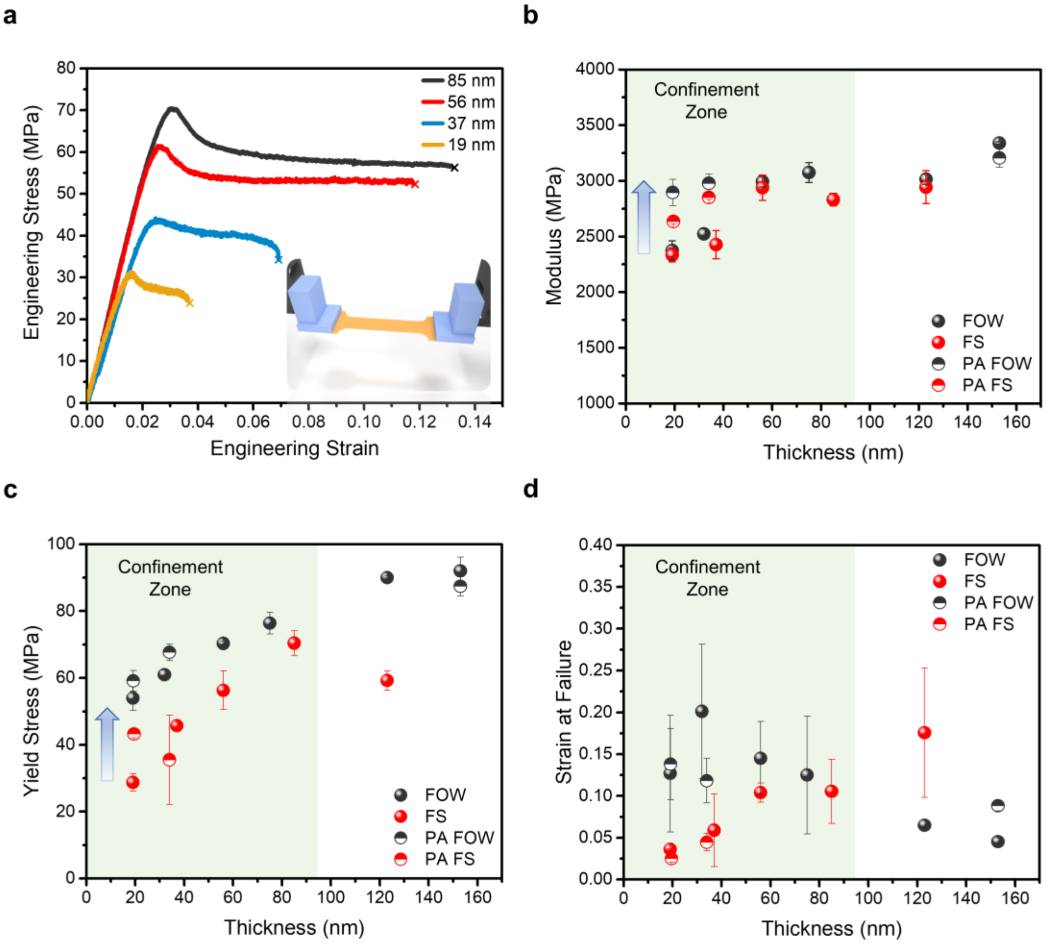

Figure S3: Comparison of 2062 kDa PS FS and FOW mechanical properties with reducing film thickness. Gage length is 8 mm. a) Representative stress vs strain profiles of FS PS from 85 nm to 19 nm. b) Elastic modulus thickness dependence of both FS and FOW PS films. Insert represents the loss of inter-entanglements upon confinement at thicknesses below the end-to-end distance of a polymer chain. c) Yield stress thickness dependence of both FS and FOW PS films. d) Strain at failure thickness dependence of both FS and FOW PS films. PA corresponds to samples characterized post vacuum annealing at 115 ˚C for 1 hr. Yield stress and strain at failure of FS PS differs with that discussed for 4 mm length films due to increased defect density exhibited by the longer gauge length of 8 mm. Error bars represent the standard deviation of the characterized mechanical properties.

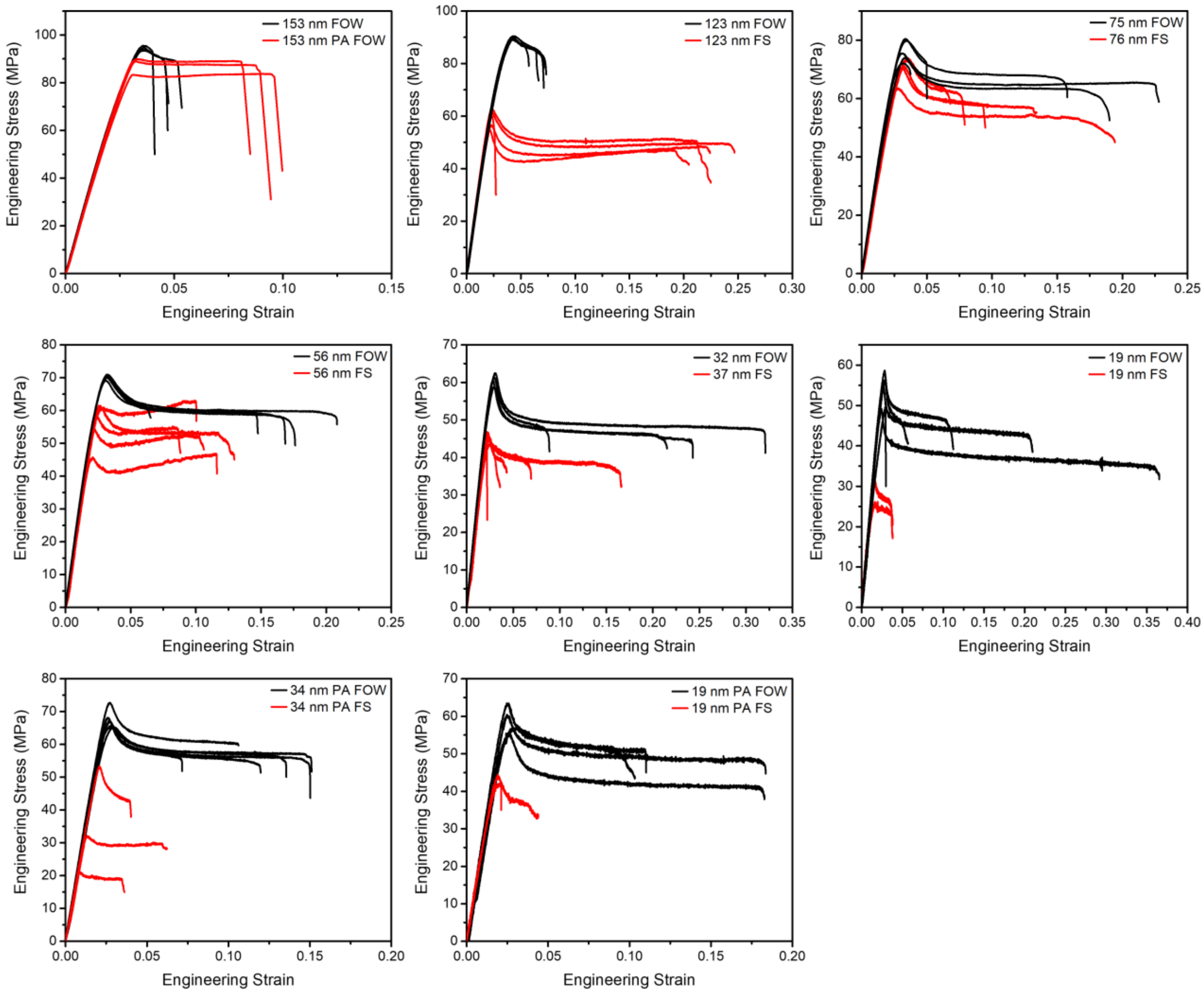

Figure S4: Stress - strain profiles of 2062 kDa PS from 153 to 19 nm. PA corresponds to post annealing at 115 ˚C for 1 hour under vacuum and slow cooled. Gage length of all films is 8 mm.

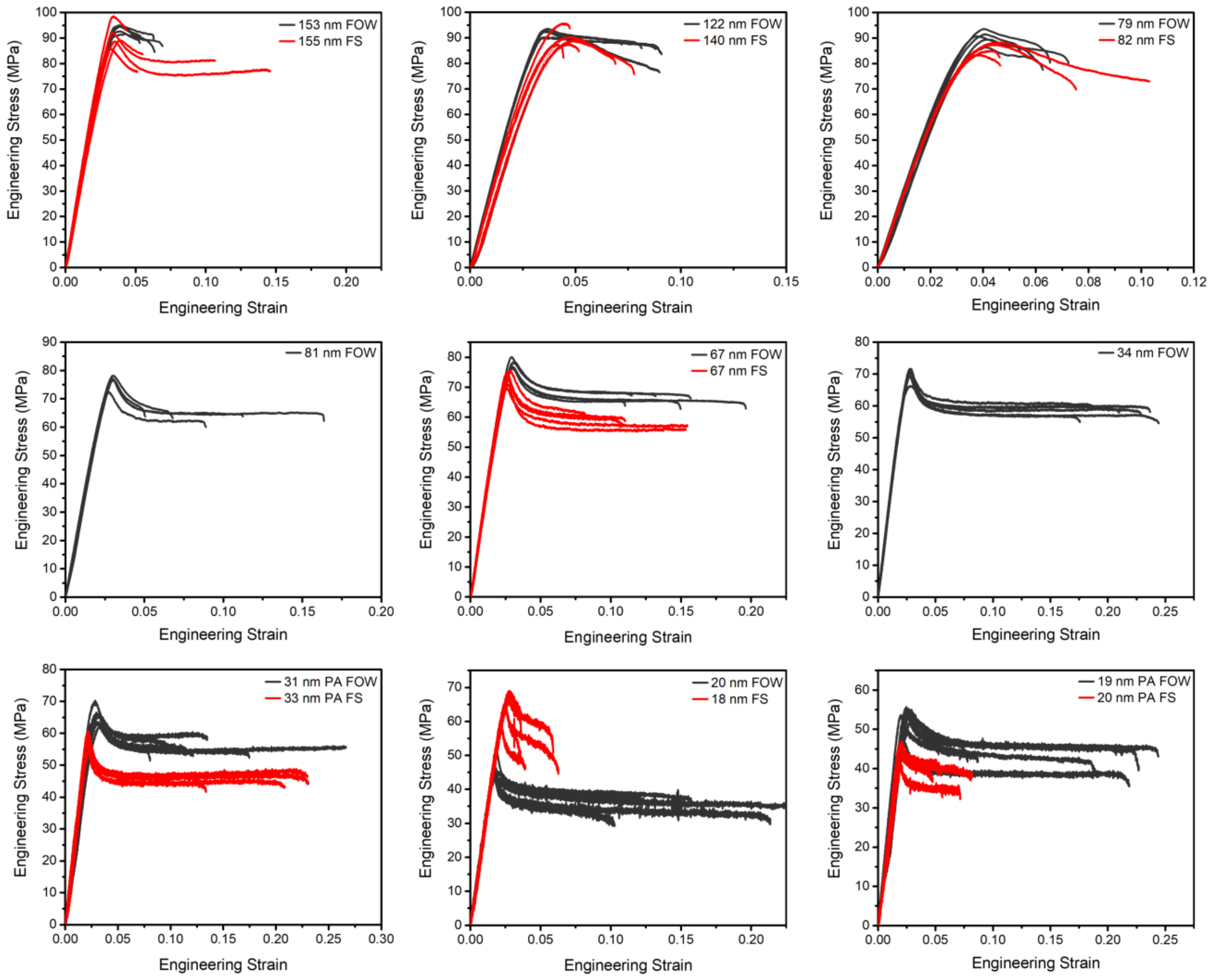

Figure S5: Stress - strain profiles of 2062 kDa PS from 155 to 19 nm. PA corresponds to post annealing at 115 ˚C for 1 hour under vacuum and slow cooled. Gage length of all films is 4 mm.

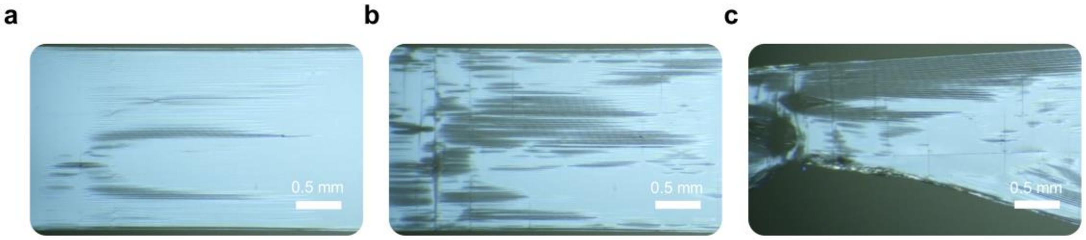

Figure S6: Optical images of 19 nm FS PS with tensile strain of a) 1.6%, b) 2.3%, and c) 3.5%. Gage length is 8 mm.

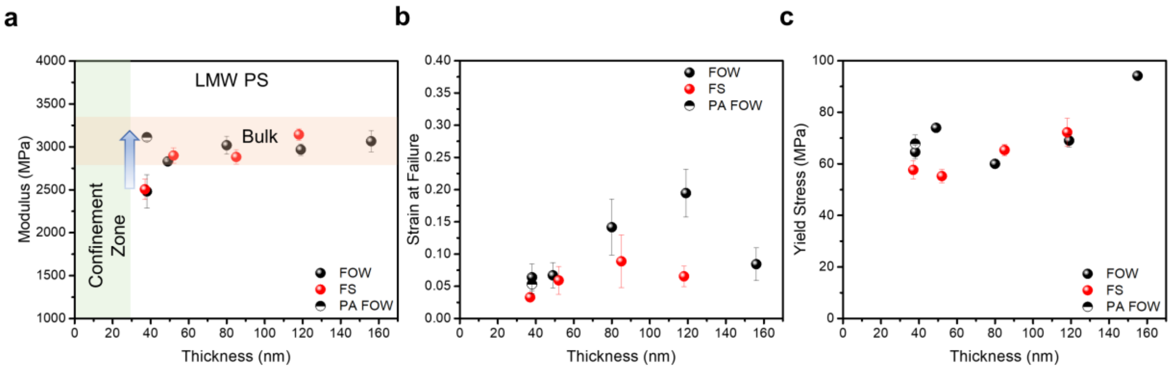

Figure S7: a) Modulus b) strain at failure and c) yield stress thickness dependence of 183 kDa PS using both FS and FOW techniques. PA corresponds to samples characterized post vacuum annealing at 115 ˚C for 1 hr. Error bars represent the standard deviation of the characterized mechanical properties.

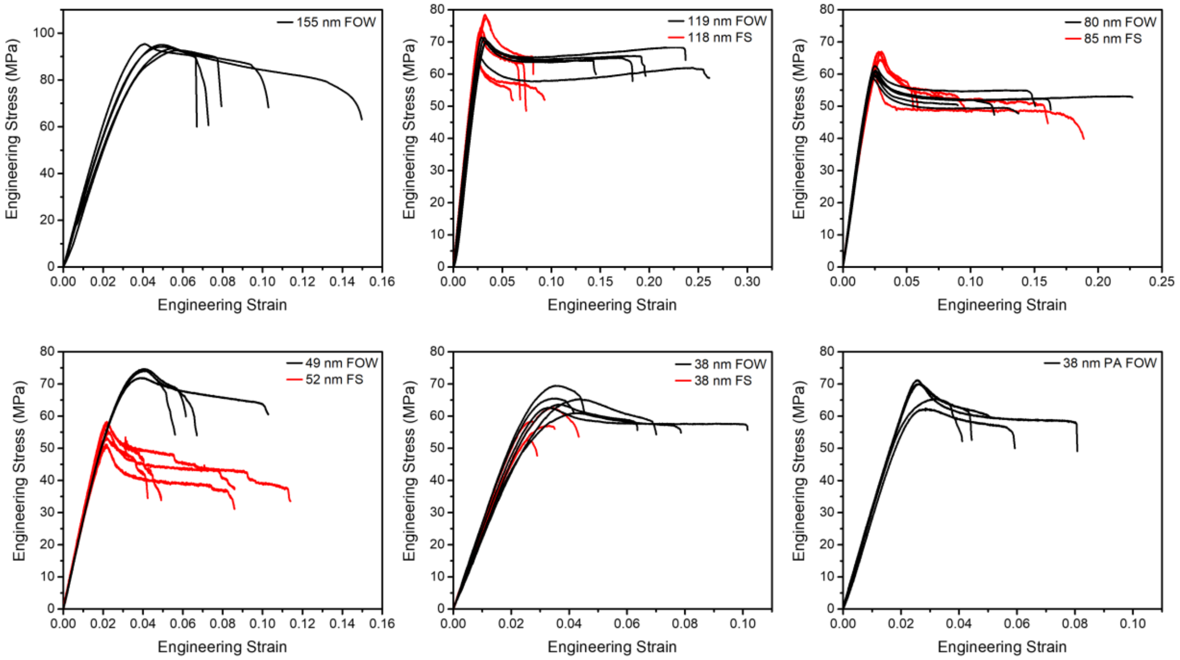

Figure S8: Stress - strain profiles of 183 kDa PS from 155 to 38 nm. PA corresponds to post annealing at 115 ˚C for 1 hour under vacuum and slow cooled.

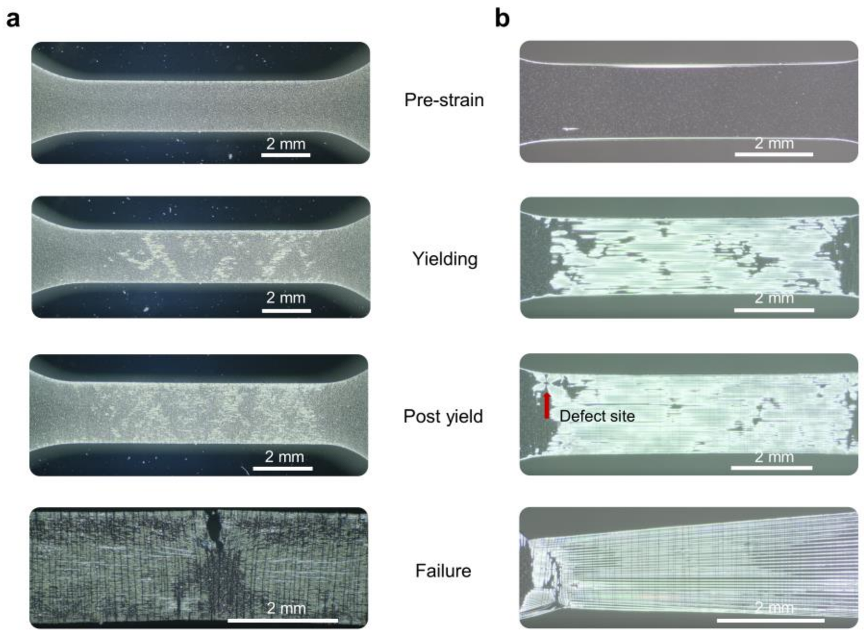

Figure S9: Optical microscopy images of 85 nm PS throughout a) on water and b) free-standing tensile strain. The free-standing film fails at the site of a defect seen in the upper left of the post yield image.

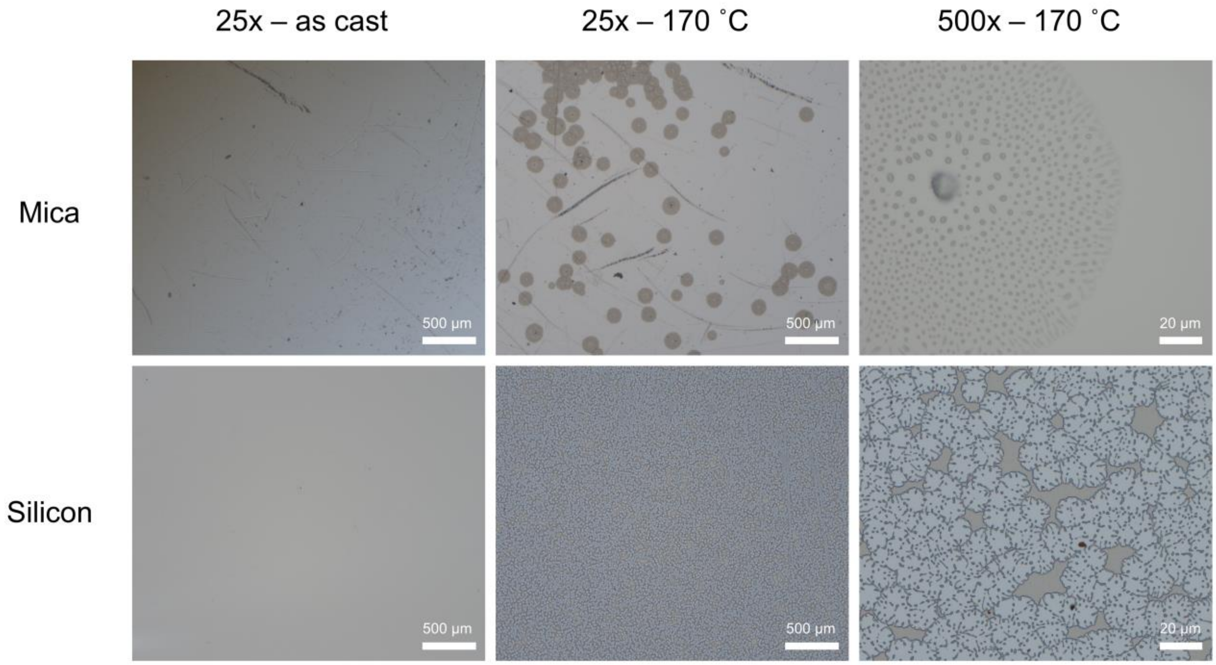

Figure S10: Optical microscopy images of polystyrene spun cast onto both mica and Si substrates followed by annealing at 170 ˚C for 25 minutes, resulting in de-wetting of the film.

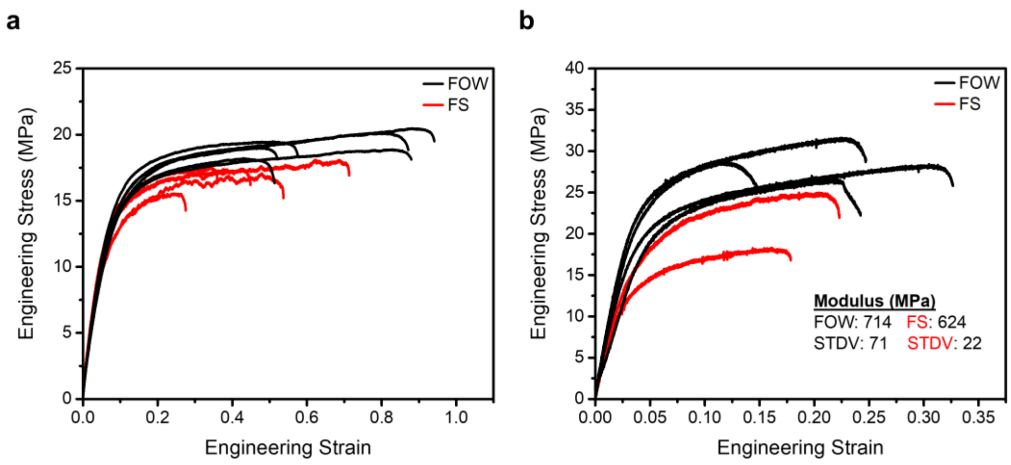

Figure S11: a) Representative stress vs strain profiles of 105 nm P3HT for both FOW and FS tensile test. b) Representative stress vs strain profiles of 75 nm DPP-TT for both FOW and FS tensile test. Insert shows the calculated modulus and standard deviation of the profiles shown.

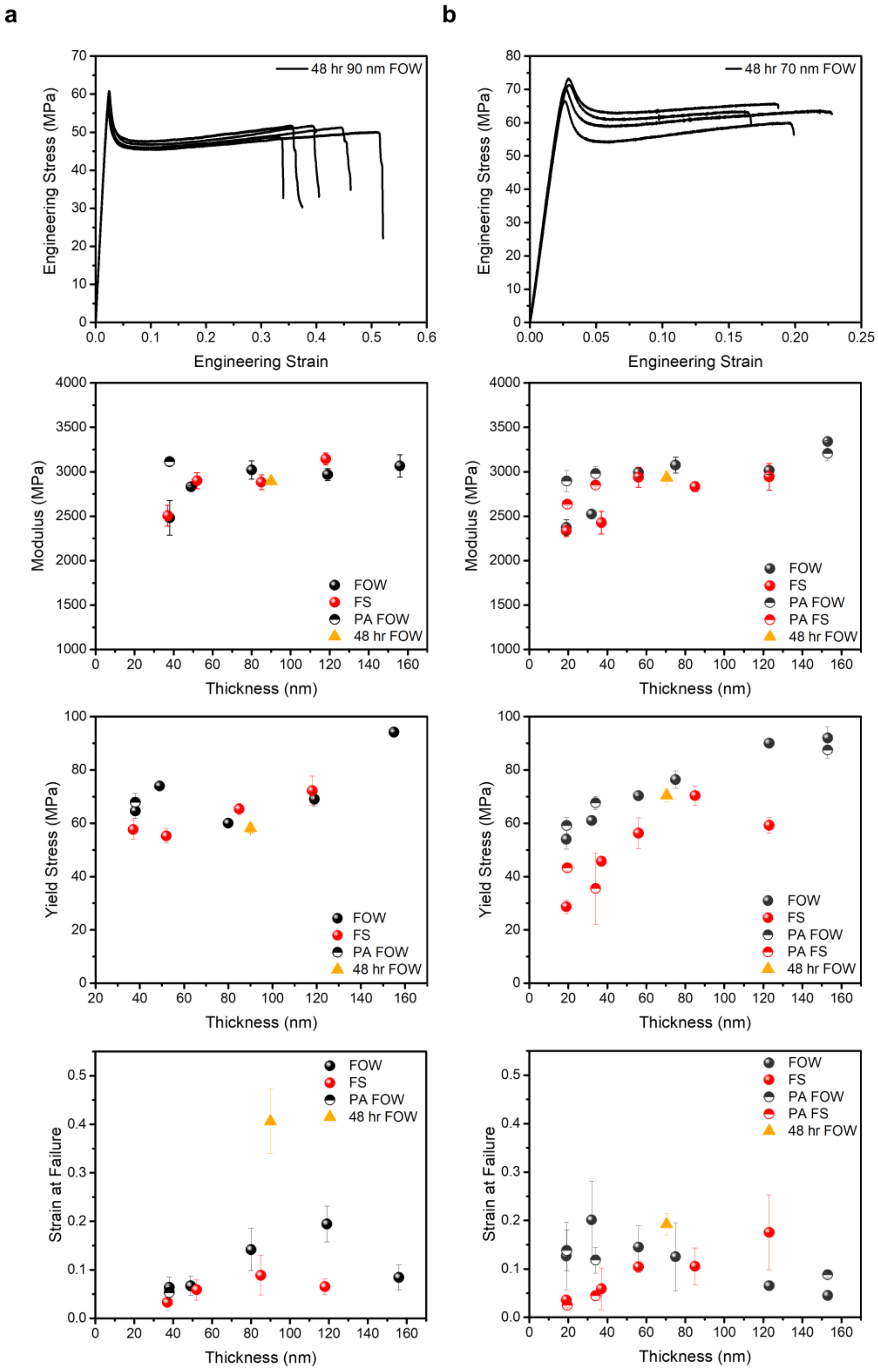

Figure S12: Measured mechanical properties of a) 90 nm 183 kDa PS and b) 70 nm 1000 kDa PS after floating on water for 48 hours compared to original tensile measurements. PA corresponds to samples characterized post vacuum annealing at 115 ˚C for 1 hr. Error bars represent the standard deviation of the characterized mechanical properties.

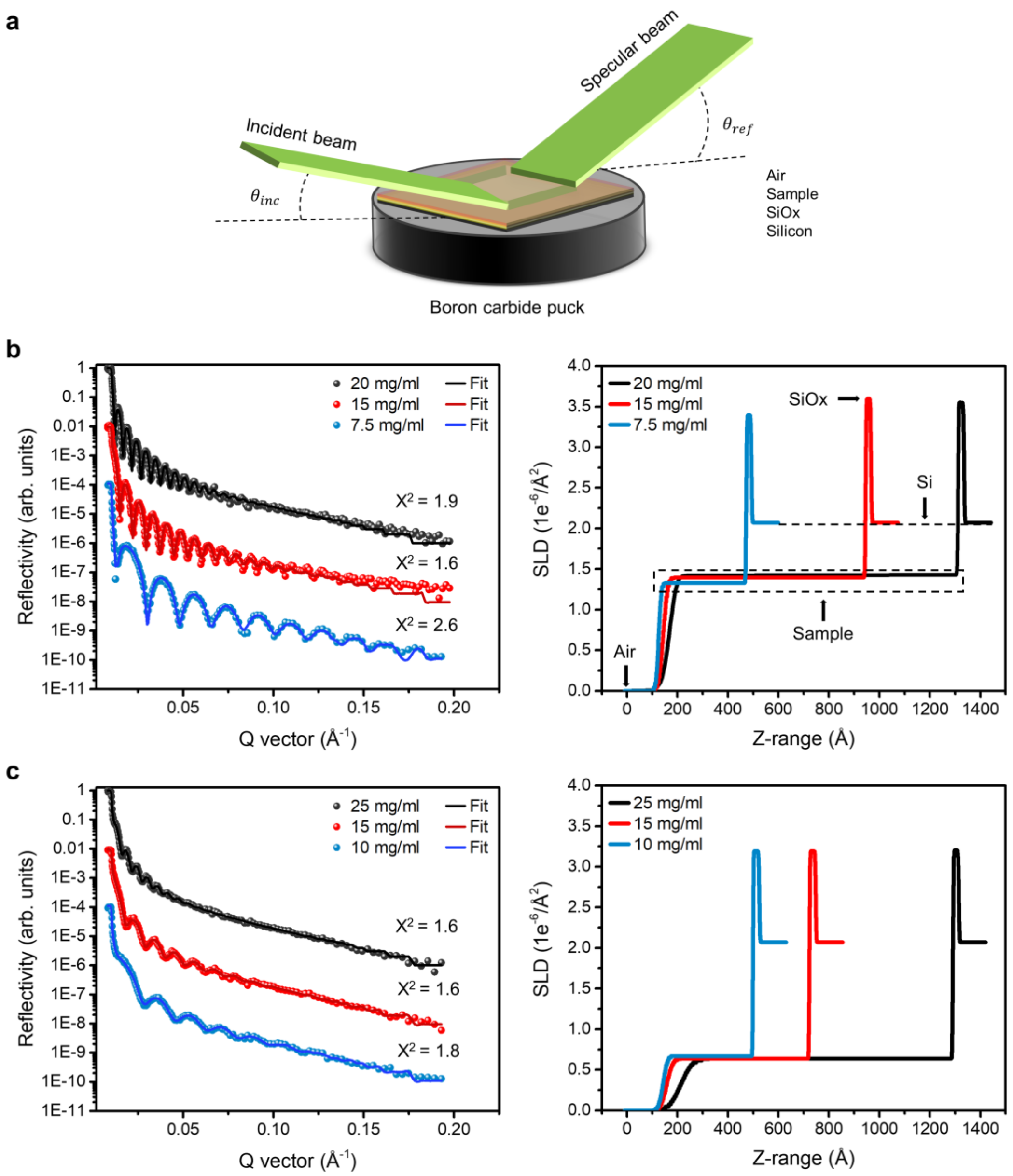

Figure S13: a) Representative illustration of the Neutron reflectivity measurement of a dry polymer sample and its associated underlying layers. Reflectivity vs. wave vector and SLD profile for b) dry PS and c) P3HT films of varying thickness. The red and blue curves in each reflectivity plot are shifted downwards by two and four orders of magnitude, respectively.

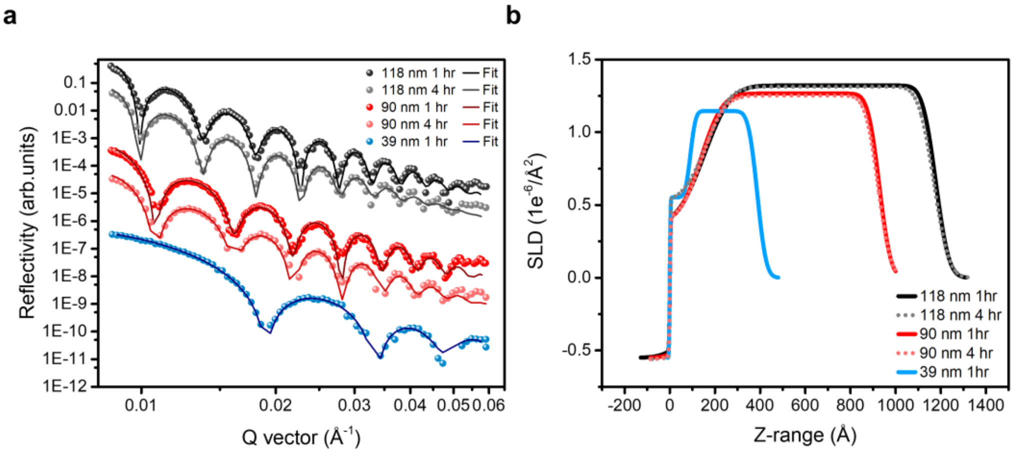

Figure S14: Time dependent neutron reflectivity a) fittings and b) SLD profile of 183 kDa PS floating on DI-water for 1 and 4 hours.

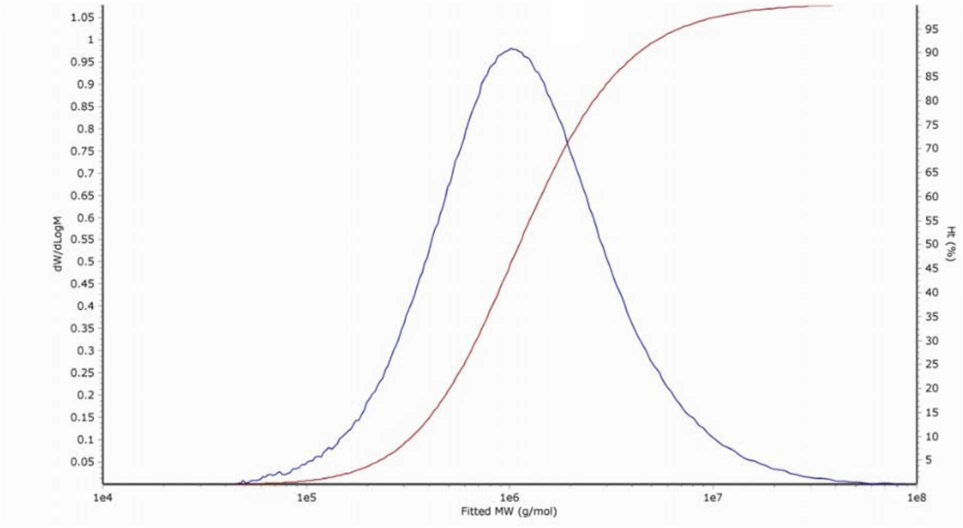

Figure S15: Molecular weight distribution of 2062 kDa PS as measured by high temperature GPC.

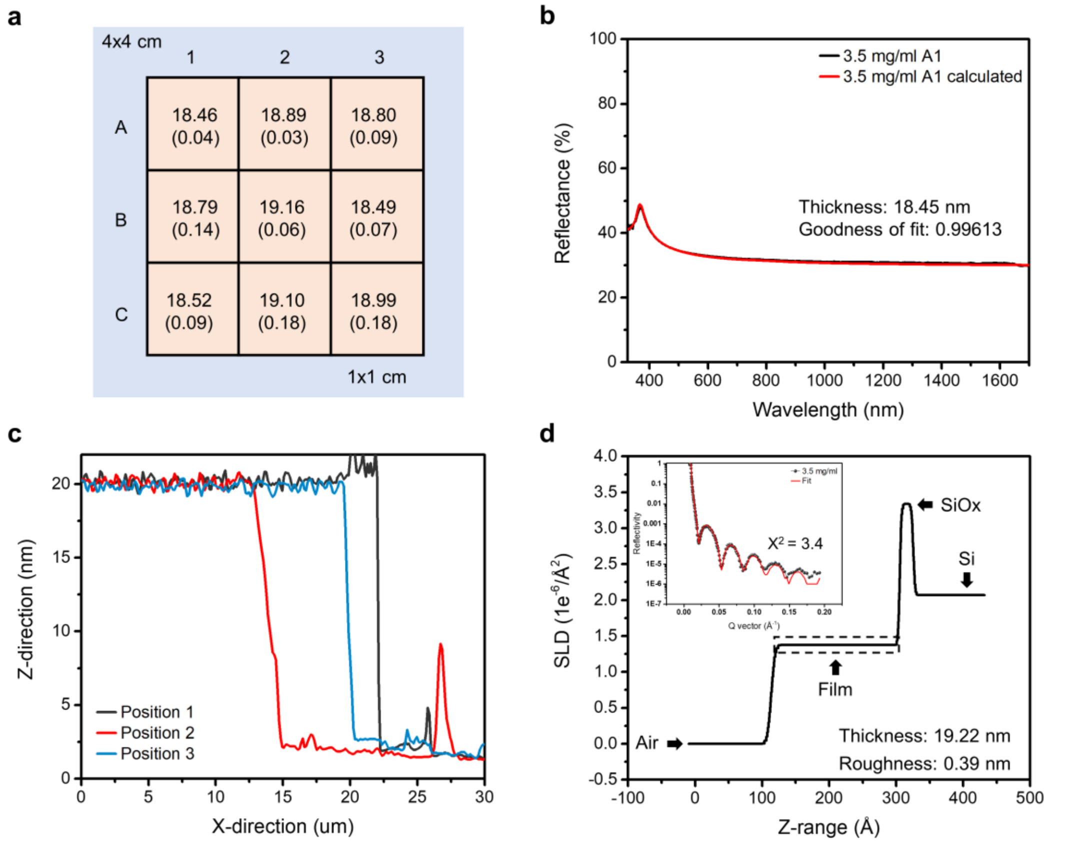

Figure S16: Film uniformity of "19" nm PS. a) Measured thickness of 1x1 cm coordinates within a parent 4x4 cm film using interferometry. Standard deviation is in parenthesis and is obtained from three separate measurements and locations within each coordinate. b) Example fitting of interferometer reflectance data of coordinate A1. c) Thickness verification using AFM within coordinate A1, 18.52 nm with a STDEV of 0.23 nm. d) Neutron reflectometry of a separate 5x5 cm PS film, without PSS release layer, demonstrating an average thickness of 19.22 nm and a roughness of 0.39 nm. Insert is of reflectometry fitting quality.

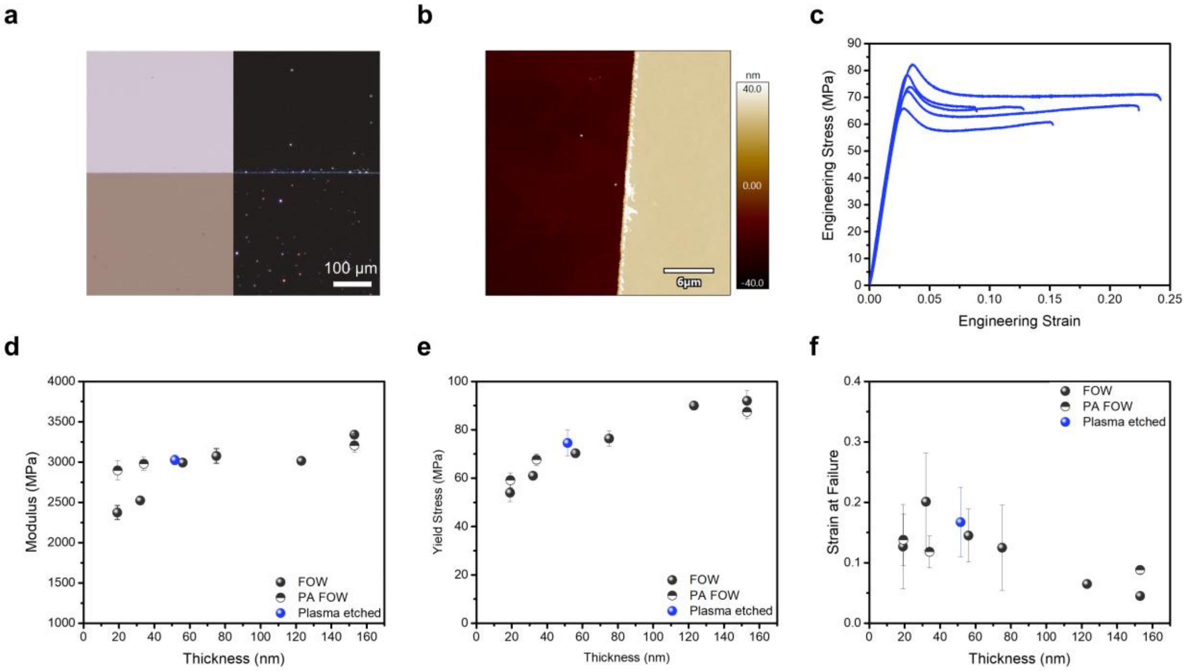

Figure S17: Plasma etched 52 nm PS film mechanical properties and comparison to the laser etched process. a) Bright and dark field optical microscopy images of plasma etched PS floated onto clean silicon. b) AFM height image of film edge. c) Stress-strain profile of five consecutive FOW tensile measurements. d-f) Modulus, yield stress, and strain at failure comparison of 52 nm plasma etched PS to that of the laser etched films. Error bars represent the standard deviation of the characterized mechanical properties.

Supplemental Tables

Table S1: Comparison of known methods for measuring the mechanical response of free-standing polymeric thin films.

Experimental technique	Current capabilities	Disadvantages
Nanobubble inflation	a. Viscoelastic response of films down to 3 nm<br>b. Environmental control	a. Large scale deformation mechanics are not obtainable (micron scale)
Camphor release	a. PC thin film mechanics down to 100 nm<br>b. Full stress-strain profile	a. Transfer process takes 24+ hours<br>b. Sub-100 nm films may not be feasible
TUFF	a. PS thin film mechanics down to 31 nm<br>b. Full stress-strain profile	a. Currently limited to 31 nm<br>b. Full yielding behavior not observed
Guide Frame	a. PS thin film mechanics down to 45 nm<br>b. Full stress-strain profile	a. Limited to 45 nm with relatively low gauge L:W ratio of 0.5<br>b. 45 nm film shows edge defects
SMART*	a. PS thin film mechanics *sub-20 nm*<br>b. Organic semiconductor mechanics *sub-100 nm*<br>c. Full stress-strain profile with *complete yielding behavior*	

*Method employed in this report

Table S2: LIQREF fitting results for 183 kDa PS and P3HT using the Amoeba/Nelder-Mead algorithm engine provided by ORNL

Polymer	Concentration (mg/ml)	Top layer thickness (Å)	Surface roughness (Å)	Top layer SLD ($1e^{-6}$/Å^2)	Sublayer thickness (Å)	Sublayer roughness (Å)	Sublayer SLD ($1e^{-6}$/Å^2)
**PS**	20	1026.0	46.1	1.321	151.4	93.5	0.361
	15	789.1	40.0	1.266	138.4	74.8	0.385
	7.5	296.3	31.0	1.146	91.3	18.4	0.552
**P3HT**	25	1016.0	43.5	0.622	75.8	60.2	0.071
	15	494.9	15.0	0.581	68.6	55.0	0.173
	10	300.7	14.0	0.558	59.3	50.4	0.192
