# Supplementary material for: SMART transfer method to directly compare the mechanical response of water-supported and free-standing ultrathin polymeric films
Source: Nat Commun. 2021 Apr 20;12:2347. doi: 10.1038/s41467-021-22473-w (PMC8058343; doi:10.1038/s41467-021-22473-w)
Supplement: Supplementary file 2 — Description of Additional Supplementary Files [file 41467_2021_22473_MOESM2_ESM.pdf]

Description of Additional Supplementary Files

File Name: Supplementary Movie 1
Description: Vertical lift of a 60 nm PS film off a water surface and subsequent failure.

File Name: Supplementary Movie 2
Description: : Smart process used to obtain and characterize a 34 nm freestanding PS film with gauge length of 8 mm.

File Name: Supplementary Movie 3
Description: : Free-standing tensile test of 19 nm polystyrene with a gauge length of 4 mm.
